# Children With Mathematical Learning Difficulties Are Sluggish in Disengaging Attention

**DOI:** 10.3389/fpsyg.2019.00932

**Published:** 2019-05-09

**Authors:** Xiaoxian Zhang, Wanlu Fu, Licheng Xue, Jing Zhao, Zhiguo Wang

**Affiliations:** ^1^School of Education, Hangzhou Normal University, Hangzhou, China; ^2^Institute of Psychological Sciences, Hangzhou Normal University, Hangzhou, China; ^3^Zhejiang Key Laboratory for Research in Assessment of Cognitive Impairments, Hangzhou, China; ^4^SR Research, Ottawa, ON, Canada

**Keywords:** attention, mathematical learning difficulty, inhibition of return, Posner cueing task, attentional disengagement

## Abstract

Mathematical learning difficulties (MLD) refer to a variety of deficits in math skills, typically pertaining to the domains of arithmetic and problem solving. The present study examined the time course of attentional orienting in MLD children with a spatial cueing task, by parametrically manipulating the cue-target onset asynchrony (CTOA). The results of Experiment 1 revealed that, in contrast to typical developing children, the inhibitory aftereffect of attentional orienting – frequently referred to as inhibition of return (IOR) – was not observed in the MLD children, even at the longest CTOA tested (800 ms). However, robust early facilitation effects were observed in the MLD children, suggesting that they have difficulties in attentional disengagement rather than attentional engagement. In a second experiment, a secondary cue was introduced to the cueing task to encourage attentional disengagement and IOR effects were observed in the MLD children. Taken together, the present experiments indicate that MLD children are sluggish in disengaging spatial attention.

## Introduction

As a milestone in cognitive development, most children acquire the ability of solving arithmetic and other mathematical problems in just a few years of formal education (Geary, 1996). About 6–14% of children,^1^ however, suffer from mathematical learning difficulties (MLD) that emerge despite of adequate intelligence, learning opportunities, and a normal sociocultural environment (Barbaresi et al., 2005). Previous studies have shown that individuals with MLD have deficits in visuospatial working memory (e.g., McLean and Hitch, 1999; Swanson and Sachse-Lee, 2001; Jarvis and Gathercole, 2003; Holmes and Adams, 2006; Bull et al., 2008) and executive functions, such as inhibition, transition, and updating (e.g., Bull and Scerif, 2001; Ashkenazi et al., 2009; Toll et al., 2011). The deficit in executive functions in MLD children, especially the inability of inhibiting task irrelevant information, is in line with the well-known fact that many MLD children display behaviors consistent with a diagnosis of attention deficit hyperactivity disorder (ADHD, e.g., Shalev et al., 1995). For the alerting, orienting, and executive control components of the attentional network (Fan et al., 2003), it has been shown that MLD children have deficits in both alerting and executive control, but not in exogenous orienting (Ashkenazi and Henik, 2010). Furthermore, recent functional neuroimaging studies have revealed that, when performing math tasks, children with mathematical difficulties have reduced activation in the intra-parietal cortex (IPS), a node of the attentional network that is critical to orienting (e.g., Kucian et al., 2006; Mussolin et al., 2010; see Corbetta and Shulman, 2002, for an in-depth discussion). On a related note, a large portion of the MLD population have comorbid reading difficulties (e.g., Lewis et al., 1994; Barbaresi et al., 2005; von Aster and Shalev, 2007), a disorder known to involve attentional dysfunctions (e.g., Facoetti et al., 2003; Ding et al., 2016; for reviews, see Hari and Renvall, 2001; Krause, 2015). These facts all point to a close link between attentional deficits and mathematical difficulties.

After attention has been oriented to a location an inhibitory tag will be left there to discourage attention from returning. This inhibitory attentional mechanism, known as inhibition of return (IOR), is widely regarded as a major driving force of orientating toward novelty (e.g., Koch and Ullman, 1985; Klein, 1988; Klein and MacInnes, 1999). IOR is frequently examined in spatial cueing tasks (Posner, 1980), in which speeded responses are required to targets preceded by uninformative peripheral cues. The sudden onset of the peripheral cue automatically captures attention and usually speeds up the response to the target when the cue-target onset asynchrony (CTOA) is brief, i.e., early facilitation effects. IOR usually emerges at relatively long CTOAs and reveals itself in slower responses to targets at cued locations (e.g., Posner and Cohen, 1984; see Klein, 2000, for a review). The onset time of IOR is about 300 ms following cue onset (for a meta-analysis, see Samuel and Kat, 2003), however, it also varies with task difficulty (e.g., Lupiáñez et al., 1997; Lupiáñez et al., 2001; see Klein, 2000, for a discussion) and reflects the flexibility of executive control. For instance, the onset time of IOR is much later in older adults, whose executive functions are declining (e.g., Langley et al., 2011; Muiños et al., 2016; Li et al., 2018). The delayed onset of IOR is likely the result of slow (or failed) disengagement of attention from the cue (Lupiáñez et al., 2001).

As noted above, the study by Ashkenazi and Henik (2010) revealed that MLD children have deficits in executive control, which is crucial for flexible and efficient orienting. The primary goal of the present study was to examine the attentional function of MLD children with a task focusing on spatial orienting. More specifically, will MLD children show a delayed onset of IOR, i.e., show a deficit in disengaging attention (as in old adults)? Children with MLD and typical developing controls (TD) were tested in the spatial cueing task, with which the CTOA was varied between 100, 200, 400, and 800 ms. Early facilitation effects were expected for short CTOAs (e.g., 100 and 200 ms), as a result of attentional capture by the peripheral cue, whereas IOR effects were expected for longer CTOAs (e.g., 400 and 800 ms). If MLD children have difficulties in disengaging attention, we expect to observe weaker (or no) IOR effects at the long CTOAs tested in the present study (e.g., 800 ms). In a second experiment, a secondary (central) cue was added to the cueing task to facilitate attentional disengagement from the cue (e.g., Briand et al., 2000; Pratt and Fischer, 2002; MacPherson et al., 2003). We expect to observe an earlier onset of IOR in MLD children if they do have deficits in attentional disengagement.

## Experiment 1: the Time Course of Attentional Orienting

### Methods

The research protocol of the present study was approved by the human research reviewing committee at the Institute of Psychological Sciences, Hangzhou Normal University. Written informed consents were obtained from the parents or other legal guardians of the children who participated in the present study.

#### Participants

In the present study, a total of 224 third-grade children from a local elementary school were screened for MLD. All children were native Mandarin speakers, and all had normal or corrected-to-normal visual acuity. According to their school records, none of them was socio-culturally disadvantaged, or had behavioral or neuropsychological conditions. They were naive with regard to the purpose of the experiments and had not participated in other psychological studies. The majority of these children were 9-year old, old enough to have well-developed capacity in exogenous attentional orienting (e.g., Akhtar and Enns, 1989; Trick and Enns, 1998). According to the end-of-year math exam results, these third-grade children had obvious individual variability in terms of mathematical performance.

Measures that are specifically designed to diagnose MLD are not available and most researchers rely on achievement tests, often in combination with measures of intelligence (IQ). In the present study, the Standard Combined Raven’s Test (CRT; Li and Chen, 1989) was used to assess the non-verbal intelligence of the children. To be included in the MLD group, a child needs to have normal intelligence, scored below the 20th percentile on the most recent end-of-year math examination (e.g., Geary et al., 2000), and have been reported by his/her math teachers as having severe and persistent math learning difficulties (Passolunghi and Mammarella, 2012). Based on these criteria, 14 children were included in the MLD group (11 girls and 3 boys; age range: 8.3–9.5 years). The control group consisted of 15 typically developing (TD) children (10 girls and 5 boys; age range: 8.5–9.5 years), randomly selected from the non-MLD children. The TD group was age-matched to the MLD group (see Table 1), Welch’s *t*-test, *t*(27) = 0.236, *p* = 0.815, Cohen’s *d* = 0.088. The MLD group scored lower on the non-verbal intelligence test (CRT), *t*(27) = 5.245, *p* < 0.001, Cohen’s *d* = 1.936, but their non-verbal IQ was all in the normal range. The scores on the most recent math exam were lower in the MLD group, *t*(27) = 15.09, *p* < 0.001, Cohen’s *d* = 3.277. As noted above, no standardized diagnostic tool is available for mathematical learning disability. We would like to explicitly acknowledge that “MLD” in the present paper refers to “mathematical learning difficulty” instead of “mathematical learning disability.”

**Table 1:** Means and SDs of the non-verbal IQ and mathematical scores in the MLD and TD children.

	*N*	Age (years)	Non-verbal IQ	Math score
TD	15	9.08 (0.29)	122.47 (11.14)	96.87 (2.69)
MLD	14	9.11 (0.34)	104.21 (7.34)	74.75 (9.16)

#### Material and Apparatus

The classic Posner cueing task (Posner and Cohen, 1984) was adopted to assess the early attentional facilitation and the later IOR effect following attentional orienting. Stimuli were presented on a 19-inch NESO CRT monitor. Data registration and stimulus presentation were controlled by a Windows 7 PC, running scripts written in Python. Eye movements were monitored with a desktop mounted EyeLink^®^ 1000 eye-tracker (SR Research, Mississauga, ON, Canada). The tracking accuracy of the eye tracker was reported to be 0.25° or better, and the participant’s gaze position was sampled at 1000 Hz.

**FIGURE 1:**
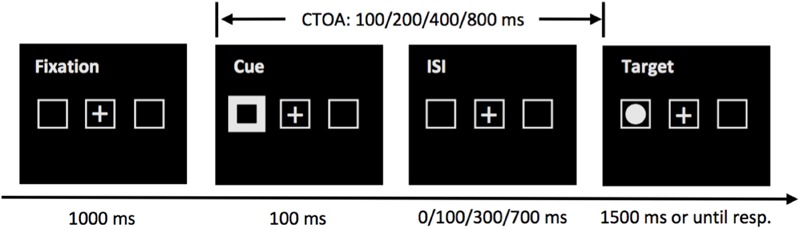
Sequence of events in a sample trial of the cueing task in Experiment 1. At the beginning of each trial, the tracking accuracy of the tracker was examined by performing a drift-check. Then, a fixation cross and three placeholders appeared on the screen, followed by a peripheral cue which was visible for 100 ms. The target was presented immediately, or 100, 300, or 700 ms later, giving four possible cue-target onset asynchronies (CTOAs): 100, 200, 400, and 800 ms. The target remained visible for a maximum of 1500 ms or until a speeded response was issued. For illustration purpose only, the stimuli are not drawn to scale.

The experiment took place in a quiet room. The participants were seated in front of a computer monitor and the viewing distance (62 cm) was maintained with a chinrest. Two gray placeholder boxes, subtending 1.80 × 1.80° visual angle, were placed on the horizontal meridian of the screen, 9.0° from a central fixation cross (see Figure 1). The cue was implemented as the brightening and thickening of one of the peripheral boxes. The target was a bright, filled circle, with a diameter of 1.0°.

#### Task Procedure

The sequence of events of a sample trial in the cueing task is presented in Figure 1. A drift-check (not shown in Figure 1) was performed at the beginning of each trial to check the tracking accuracy of the tracker. The participant was required to look at a visual target on the screen and the experimenter pressed the space bar on the EyeLink Host PC keyboard to accept a stable gaze. Successful drift-check was achieved when the participant’s gaze position was within 2° from the visual target. Then, two gray placeholder boxes were presented for 1000 ms on the screen, together with a fixation cross at the screen center. Then the cue appeared at the left or right peripheral boxes for 100 ms. The target was presented 100, 200, 400, or 800 ms later to examine the early attentional facilitation effect and the later IOR effect. The target appeared in one of the two peripheral boxes, and the participants were asked to respond with the space bar as quickly and as accurately as possible on a standard QWERTY keyboard, and the target remained visible for 1500 ms, or until the participant made a response. To discourage anticipatory responses, the target did not appear on 25% of the trials. The target could appear in the cued box (validly cued) or in the box opposite to the cue (invalidly cued), with equal probabilities.

The cueing task consisted of four blocks of 40 trials. The eye-tracker was calibrated with a standard nine-point calibration routine at the beginning of each block, or whenever a break was required by the participant. The participants were explicitly instructed to maintain fixation at the central box throughout a trial. If fixation was broken on two successive trials, the participants were reminded by the experimenter to keep looking at the central box and not to move their eyes.

The early facilitation effect is typically attributed to attentional capture by the cue and is manifested in faster RTs to validly cued compared to invalidly cued targets, whereas IOR is manifested in slower RTs to validly cued compared to invalidly cued targets.

### Results

To discourage anticipatory responses, 25% of the trials were catch trials. An ANOVA on the false alarm rates, with variables group (MLD vs. TD) and CTOA, revealed no significant effect, all *F* < 1. The false alarm rates for the MLD and TD children were 3.79 and 3.13%, respectively. An ANOVA on the miss rates revealed only a main effect of group, *F*(1,27) = 8.08, *p* = 0.008, $\eta_{p}^{2}$ = 0.230; the miss rates for the MLD and TD children were 2.01 and 0.36%, respectively. No other effect was significant.

The active engagement of the oculomotor system may invoke untoward motoric effects that cannot be attributed to attention (e.g., Hilchey et al., 2014), so trials during which eye movements occurred were excluded from the analysis. These trials accounted for 15.90 and 13.02% of the trials (including catch trials) in the MLD and TD children, respectively. An ANOVA on the proportions of trials excluded due to eye movements, with variables group (MLD vs. TD) and CTOA, revealed no main effect for group, *F* < 1. There was a main effect of CTOA, *F*(3,81) = 3.67, *p* = 0.016, $\eta_{p}^{2}$ = 0.120; the eyes were less likely to move away from the central box at the long CTOAs. The temporal expectation for the target strengthens as the CTOA increases and consequently, the onset of the cue is less likely capture the eyes (i.e., oculomotor capture; Theeuwes et al., 1998). The two-way interaction was not significant, *F* < 1.

The RTs from the non-catch trials were cleaned based on the number of trials in each experimental cell of each participant, following the criteria given in Van Selst and Jolicoeur (1994, Table 4). This outlier removal procedure effectively controls the impact of the number of successfully completed trials in different experimental cells. It has been widely adopted by researchers in the field, with various automated tools (e.g., R packages) freely available online. This procedure excluded only a small proportion of the trials, 3.06%, 3.22% for the MLD and TD children, respectively. An ANOVA on the proportion of trials excluded due to this procedure revealed no main effect or interaction.

**FIGURE 2:**
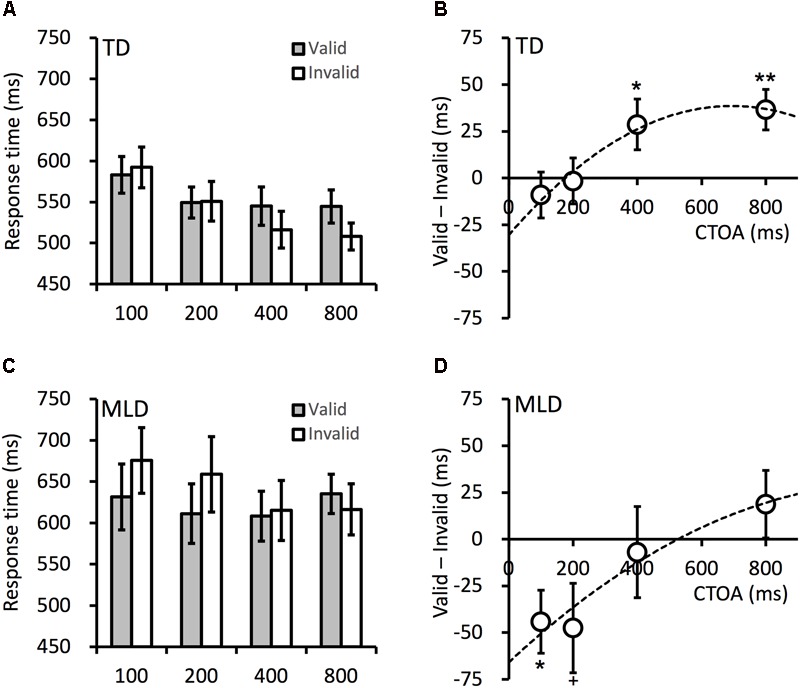
Mean target RTs in the TD **(A)** and MLD **(C)** children. The cueing effects in the TD **(B)** and MLD **(D)** children. CTOA is the time interval (in milliseconds) between cue onset and target onset (see Figure 1 for an illustration). Dashed lines are polynomial fittings of the cueing effects against CTOA. Error bars denote ± 1 SEM. ^+^*p* < 0.06, ^∗^*p* < 0.05, ^∗∗^*p* < 0.01.

The attentional effects in cueing tasks are typically quantified with the RT difference between validly and invalidly cued targets: the early attentional facilitation effect is manifested in faster RTs to validly cued targets, whereas the later IOR effect is manifested in slower RTs to validly cued targets. The mean RTs in all conditions are presented in Figure 2. To examine whether these attentional effects were impaired in children with MLD, the RTs were submitted to an ANOVA, with variables group (MLD vs. TD), cueing (valid vs. invalid), and CTOA (100, 200, 400, or 800 ms). The results revealed significant main effects for group, *F*(1,27) = 5.213, *p* = 0.031, $\eta_{p}^{2}$ = 0.162, and CTOA, *F*(3,81) = 11.00, *p* < 0.001, $\eta_{p}^{2}$ = 0.290. The RTs were longer in the MLD group, and generally decreased as the CTOA increased. The main effect of cueing was not significant, *F*(1,27) = 0.097, *p* = 0.757, $\eta_{p}^{2}$ = 0.004. This was not unexpected as facilitation effects are typically observed at short CTOAs, whereas IOR effects are expected for longer CTOAs. A significant two-way interaction occurred between cueing and CTOA, *F*(3,81) = 8.135, *p* < 0.001, $\eta_{p}^{2}$ = 0.232, suggesting the cueing effect varied across the CTOAs. The two-way interactions between cueing and group was marginal, *F*(1,27) = 4.13, *p* = 0.052, $\eta_{p}^{2}$ = 0.133; the cueing effects were more positive in the TD group. The interaction between CTOA and group, *F*(3,81) = 1.037, *p* = 0.381, $\eta_{p}^{2}$ = 0.037, and the three-way interaction, *F*(3,81) = 0.389, *p* = 0.761, $\eta_{p}^{2}$ = 0.014, were not significant. When the non-verbal IQ of the children was included as a covariate in the ANOVA, the overall pattern of the results was the same as that reported here, expect that the main effect of group is no longer significant.

The time course of attentional orienting in cueing tasks is characterized by facilitation effects at short CTOAs and IOR effects at longer CTOAs (>300 ms). As shown in Figure 2, this overall pattern was present in both the MLD and TD children. To examine the cueing effect at each CTOA, planned contrasts were performed to compare the RTs between validly and invalidly cued targets. For TD children, no facilitation effect was observed, all *t* < 1, all *p* > 0.450, but significant IOR effects were observed at the 400-ms CTOA, *t*(14) = 2.187, *p* = 0.046, *dz* = 0.565, and the 800-ms CTOA, *t*(14) = 3.497, *p* = 0.004, *dz* = 0.903. For MLD children, facilitation effects were observed at the 100-ms CTOA, *t*(13) = 2.717, *p* = 0.018, *dz* = 0.726, and the 200-ms CTOA, *t*(13) = 2.061, *p* = 0.060, *dz* = 0.551. The IOR effect, however, was not observed at the longer CTOAs (400 ms and 800 ms) tested in the present experiment, all *t* < 1.10, all *p* > 0.300.

### Discussion

The cueing effects are presented in the right column in Figure 2. The cueing effect, overall, was more positive (i.e., leaning toward IOR) in TD children. The MLD children did not show any IOR effect, event at the longest CTOA (800 ms) tested in the present study, whereas TD children showed strong IOR effects at the 400- and 800-ms CTOAs. The onset time of IOR can be examined by fitting the cueing effects (group means) against the CTOAs with a quadratic polynomial function (Li et al., 2018). The onset time of IOR is the CTOA at which the cueing effect crosses 0-ms. In the present experiment, the onset time of IOR was 480 and 175 ms in the MLD and TD children, respectively. As shown in Figure 2D, robust facilitation effects were observed at short CTOAs in MLD children; the MLD children had no difficulty in orienting attention to the cue. The delayed onset of IOR in MLD children was most likely the result of difficulties in disengaging attention from the cued location.

The sample size was relatively small in Experiment 1. A second set of data was collected with exactly the same screening criteria and experimental methods to address statistical power concerns. An analysis combining this additional dataset and that from Experiment 1 is presented as Supplementary Material. This combined analysis effectively increased the sample size to 35 and 34 for the TD and MLD groups, respectively. The overall pattern of the results was the same, i.e., the TD children showed the typical time course of the cueing effect whereas only facilitation effects were observed in the MLD children (see Supplementary Material for details).

The RTs were longer for the MLD children compared to the TD children. One possible explanation for this observation is that the MLD children have lower processing speed compared to healthy controls (e.g., Geary et al., 2011). However, a difference in processing speed cannot explain the onset delay of IOR in the MLD children. The cueing effects were estimated with the RT difference between validly and invalidly cued targets, a difference in processing speed should have been controlled for when the cueing effects were derived.

## Experiment 2: Re-Orienting Cue

The second experiment was designed to further examine the main finding of Experiment 1, i.e., children with MLD are sluggish in disengaging attention. The task was the same as that in Experiment 1, with the addition of a secondary cue, which is known to facilitate the disengagement of attention from the cued peripheral location in cueing tasks (e.g., Briand et al., 2000; Pratt and Fischer, 2002; MacPherson et al., 2003).

### Methods

#### Participants

The same group of MLD children took part in Experiment 2.

#### Material, Apparatus, and Task Procedure

**FIGURE 3:**
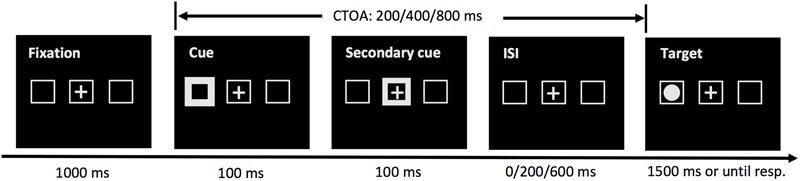
Sequence of events in a sample trial of the cueing task in Experiment 2. The task was the same as that of Experiment 1, except that a secondary cue was presented to draw attention back to the screen center and three CTOAs (200, 400, and 800 ms) were examined.

The cueing task tested in Experiment 2 was the same as that in Experiment 1, except that a secondary cue was presented at the center of the screen for 100 ms, immediately following the presentation of the peripheral cue (see Figure 3). To accommodate the secondary cue, the 100-ms CTOA was not tested.

Experiment 1 had shown that the onset time of IOR in MLD children was much later compared to that in TD children. No reliable IOR effect was observed even at the longest CTOA tested (800 ms) in Experiment 1. A secondary cued is known to facilitate the disengagement of attention from the cued location. With a secondary cue, we expected to observe IOR effect at a much shorter CTOA in Experiment 2.

### Results

The false alarm rate in Experiment 2 was 4.46% and the target was rarely missed (2. 08%). On average, eye movements were detected on 12.87% of the trials (including catch trials). As in Experiment 1, these trials were excluded from analysis. The RTs were cleaned with the same protocol as Experiment 1; this procedure removed only 3.87% of the non-catch trials.

**FIGURE 4:**
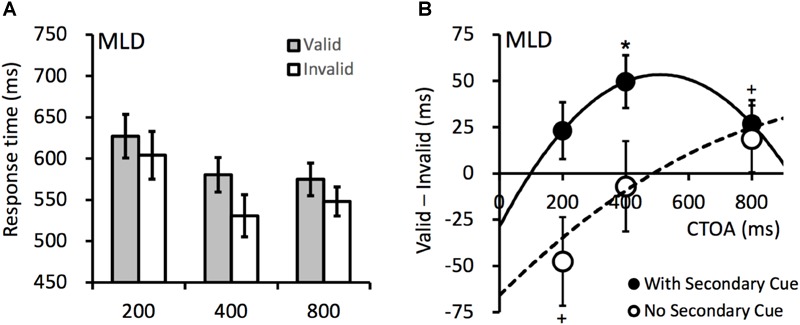
**(A)** Mean RTs (ms) in Experiment 2. **(B)** The cueing effects observed when the secondary cue was present (filled circles, Experiment 2) or absent (empty circles, Experiment 1). The solid and dashed lines are polynomial fittings of the cueing effects against CTOA. Error bars denote ± 1 SEM. ^+^*p* < 0.06, ^∗^*p* < 0.05.

The mean RTs are presented in Figure 4A. To examine the effect of the secondary cue, an ANOVA was performed on the RTs from both Experiment 1 (without secondary cue) and Experiment 2 (with secondary cue), with variables secondary cue (with vs. without), CTOA (200, 400, and 800 ms), and cueing (valid vs. invalid). The results revealed significant main effects for CTOA, *F*(2,26) = 6.441, *p* = 0.005, $\eta_{p}^{2}$ = 0.331, and secondary cue, *F*(1,13) = 11.240, *p* = 0.005, $\eta_{p}^{2}$ = 0.464. The RTs were shorter at longer CTOAs (i.e., a foreperiod effect). The RTs in Experiment 2 were generally shorter because the secondary cued may have served as a warning signal. A significant two-way interaction occurred between cueing and CTOA, *F*(2,26) = 4.072, *p* = 0.029, $\eta_{p}^{2}$ = 0.239, because the cueing effect increased with CTOA (see Figure 4B). A two-way interaction also occurred between cueing and secondary cue, *F*(1,13) = 6.872, *p* = 0.021, $\eta_{p}^{2}$ = 0.346, as the cueing effects were overall more positive when the secondary cue was presented (in Experiment 2). The three-way interaction did not reach significance, *F*(2,26) = 1.891, *p* = 0.171, $\eta_{p}^{2}$ = 0.127. For the RTs from Experiment 2, planned contrasts revealed a significant IOR effect at the 400-ms CTOA, *t*(13) = 3.611, *p* = 0.003, *dz* = 0.965, and a marginally significant IOR effect at the 800-ms CTOA, *t*(13) = 2.154, *p* = 0.051, *dz* = 0.575.

### Discussion

The cueing effects are presented in Figure 4B. The secondary cue at fixation facilitated attentional disengagement and consequently, reliable IOR effect was observed at the 400-ms CTOA (same as in the TD children). Fitting the cueing effects against the CTOA revealed that the onset time of IOR in the MLD group was 99 ms in Experiment 2 (with secondary cue), much earlier than that in Experiment 1 (no secondary cue).

The task of Experiment 2 was performed by the MLD children immediately following the completion of the cueing task of Experiment 1, or in a separate session on the following day or 3 weeks later. This less-than-optimal testing protocol was adopted for practical reasons: (a) the school gave us fairly limited time to test the students, and (b) the primary goal of the present study was to examine whether the onset of IOR is delayed in MLD children; the task of Experiment 1 had to be carried out first, so the cueing task used to examine the time course of attentional orienting would not be contaminated in any way. The RTs in Experiment 2 were generally shorter. We cannot rule out a possible practice effect, but we believe that the shorter RTs were mainly contributed to by the secondary cue, which may have served as an additional warning signal for the target. The faster RTs in Experiment 2 did not comprise our conclusions in any way. The primary measure of interest to us was the cueing effect, i.e., the RT difference between validly and invalidly cued targets. As the RT decreases one would expect the cueing effect to also decrease. However, as supported by the two-way interaction between cueing and secondary cue, the cueing effects in Experiment 2 were overall stronger (more positive).

## General Discussion

Efficient attentional orienting is indispensable for various cognitive tasks, for instance, reading, decision making, and problem solving. The field has seen an increasing number of behavioral and neuroimaging studies suggesting that individuals with MLD may suffer from attentional dysfunctions. Using a spatial cueing task, the present study examined whether a deficit in attentional orienting exists in children with MLD. The MLD children showed facilitation effects at the 100- and 200-ms CTOAs, suggesting that they had no trouble in orienting attention toward salient spatial locations. However, in contrary to TD children, the IOR effect was absent in MLD children, even at the longest CTOA (800 ms) tested in the present study. Fitting the cueing effects against the CTOAs with a quadratic polynomial function revealed that the onset time of IOR was markedly delayed in MLD children, suggesting that MLD children have difficulties in disengaging attention. To further verify this conclusion, the MLD children were tested with a second cueing task, in which a secondary cue was presented to facilitate the disengagement of attention. An IOR effect was observed at the 400-ms CTOA, providing additional evidence that MLD children have difficulties in attentional disengagement. These findings are in line with the results of recent neuroimaging studies, which showed that MLD children had reduced activation in the intraparietal sulci area (IPS, e.g., Kucian et al., 2006; Price et al., 2007; Mussolin et al., 2010), an area closely linked to attentional shifting and IOR (Marois et al., 2000; Lepsien and Pollmann, 2002; Giesbrecht and Kingstone, 2004).

The present study was largely explorative by its nature. While the experiments presented here revealed that MLD children are sluggish in disengaging attention, it remains unclear how this attentional dysfunction impairs mathematical learning in MLD children. Previous studies have found that children with developmental dyslexia have a similar impairment in attentional orienting (e.g., Facoetti et al., 2006, 2010; Ding et al., 2016). It could be that children with various forms of learning disabilities (e.g., dyslexia) are all suffering from a domain-general deficit in attention, which leads to less efficient visual sampling and consequently, difficulties in cognitive activities, such as solving mathematical problems and reading. A recent training study showed that attentional training with video games improved the efficiency of attentional orienting in MLD adults, however, no obvious improvement in arithmetic or basic numerical processing was observed (Ashkenazi and Henik, 2012), suggesting that the attentional deficit among those with MLD and the deficits in numerical processing may arise from different sources.

Attentional training has been proven effective in improving the reading ability of dyslexic children (e.g., Facoetti et al., 2003; Franceschini et al., 2013; Gori et al., 2016). It is unclear why attentional training failed to improve the basic numerical abilities in MLD adult in Ashkenazi and Henik (2012). The participants in this study were adults; it is possible that there exists a critical period for attentional intervention among MLD individuals. It would be interesting to examine if the attentional training programs that have proven effective among dyslexic children are also helpful to MLD children.

It is important to also note that there is no generally accepted diagnostic criteria for MLD. Lewis et al. (1994) discriminated children with specific arithmetic difficulties, with combined arithmetic-and-reading difficulties, and with specific reading difficulties. Unfortunately, we did not assess the reading ability of the participants of the present study with standardized reading tests and could not discriminate if the MLD children had comorbid ADHD or reading problems. Given that MLD children may have comorbid reading difficulties or/and ADHD, it would be beneficial to study the attentional deficit in dyslexic, ADHD and, MLD children from the same population in future studies.

To summarize, the present experiments showed that the onset time of IOR among MLD children is later than typical developing children. We conclude that attentional disengagement is impaired in MLD children and would like to encourage researchers in the field to examine whether attentional training can effectively improve the mathematical ability of MLD children.

## Ethics Statement

This study was carried out in accordance with the recommendations of “the human research reviewing committee of the Institute of Psychological Sciences, Hangzhou Normal University” with written informed consent from all parents or other legal guardians of all children who participated the present study. The protocol was approved by “the human research reviewing committee of the Institute of Psychological Sciences, Hangzhou Normal University.”

## Author Contributions

All authors designed the experiments and approved the final version of the manuscript for submission. WF collected the data. XZ, WF, and JZ analyzed the data. XZ drafted the manuscript. JZ, LX, and ZW provided the critical revisions.

## Conflict of Interest Statement

The authors declare that the research was conducted in the absence of any commercial or financial relationships that could be construed as a potential conflict of interest.

## Footnotes

1 The prevalence of MLD reported in the literature depends highly on the screening criteria adopted by the researchers. For instance, Geary (2004) gave an estimation of 5–8%, whereas the number given by Lewis et al. (1994) was much lower, only 3.6%.

## References

[B1] Akhtar N. Enns J. T. (1989). Relations between convert orienting and filtering in the development of visual attention. *J. Exp. Child Psychol.* 48 315–334. 10.1016/0022-0965(89)90008-8 2794859

[B2] Ashkenazi S. Henik A. (2010). Attentional networks in developmental dyscalculia. *Behav. Brain Funct.* 6:2. 10.1186/1744-9081-6-2 20157427 PMC2821357

[B3] Ashkenazi S. Henik A. (2012). Does attentional training improve numerical processing in developmental dyscalculia? *Neuropsychology* 26 45–56. 10.1037/a0026209 22081984

[B4] Ashkenazi S. Rubinsten O. Henik A. (2009). Attention, automaticity, and developmental dyscalculia. *Neuropsychology* 23 535–540. 10.1037/a0015347 19586217

[B5] Barbaresi W. J. Katusic S. K. Collagin R. C. Weaver A. L. Jacobsen S. J. (2005). Math learning disorder: incidence in a population-based birth cohort, 1976-82, Rochester, Minn. *Ambul. Pediatr.* 5 281–289. 10.1007/s10803-008-0645-8 16167851

[B6] Briand K. A. Larrison A. L. Sereno A. B. (2000). Inhibition of return in manual and saccadic response systems. *Percept. Psychophys.* 62 1512–1524. 10.3758/BF03212152 11140175

[B7] Bull R. Epsy K. A. Wiebe S. A. (2008). Short-term memory, working memory, and executive functioning in preschoolers: longitudinal predictors of mathematical achievement at age 7 years. *Dev. Neuropsychol.* 33 205–228. 10.1080/87565640801982312 18473197 PMC2729141

[B8] Bull R. Scerif G. (2001). Executive functioning as a predictor of children’s mathematics ability: inhibition, switching, and working memory. *Dev. Neuropsychol.* 19 273–293. 10.1207/s15326942DN1903_3 11758669

[B9] Corbetta M. Shulman G. (2002). Control of goal-directed and stimulus-driven attention in the brain. *Nat. Rev. Neurosci.* 3 201–215. 10.1038/nrn755 11994752

[B10] Ding Y. Zhao J. He T. Tan Y. Zheng L. Wang Z. (2016). Selective impairment of covert attentional shift in Chinese dyslexic children. *Dyslexia* 22 362–378. 10.1002/dys.1541 27805322

[B11] Facoetti A. Luisa Lorusso M. Paganoni P. Umiltà C. Gastone Mascetti G. (2003). The role visuospatial attention in developmental dyslexia: evidence from a rehabilitation study. *Cogn. Brain Res.* 15 154–164. 10.1016/S0926-6410(02)00148-9 12429367

[B12] Facoetti A. Trussardi A. N. Ruffino M. Lorusso M. L. Cattaneo C. Galli R. (2010). Multisensory spatial attention deficits are predictive of phonological decoding skills in developmental dyslexia. *J. Cogn. Neurosci.* 22 1011–1025. 10.1162/jocn.2009.21232 19366290

[B13] Facoetti A. Zorzi M. Cestnick L. Lorusso M. L. Molteni M. Paganoni P. (2006). The relationship between visuo-spatial attention and nonword reading in developmental dyslexia. *Cogn. Neuropsychol.* 23 841–855. 10.1080/02643290500483090 21049356

[B14] Fan J. Fossella J. Sommer T. Wu Y. Posner M. I. (2003). Mapping the genetic variation of executive attention onto brain activity. *Proc. Natl. Acad. Sci. U.S.A.* 100 7406–7411. 10.1073/pnas.0732088100 12773616 PMC165888

[B15] Franceschini S. Gori S. Ruffino M. Viola S. Molteni M. Facoetti A. (2013). Action video games make dyslexic children read better. *Curr. Biol.* 23 462–466. 10.1016/j.cub.2013.01.044 23453956

[B16] Geary D. C. (1996). *Children’s Mathematical Development: Research and Practical Applications*. Washington, DC: American Psychological Association.

[B17] Geary D. C. (2004). Mathematics and learning disabilities. *J. Learn. Disabil.* 37 4–15. 10.1177/00222194040370010201 15493463

[B18] Geary D. C. Hamson C. O. Hoard M. K. (2000). Numerical and arithmetical cognition: a longitudinal study of process and concept deficits in children with learning disability. *J. Exp. Child Psychol.* 77 236–263. 10.1006/jeep.2000.2561 11023658

[B19] Geary D. C. Hoard M. K. Bailey D. H. (2011). Fact retrieval deficits in low achieving children and children with mathematical learning disability. *J. Learn. Disabil.* 45 291–307. 10.1177/0022219410392046 21252374 PMC3163113

[B20] Giesbrecht B. Kingstone A. (2004). Right hemisphere involvement in the attentional blink: evidence from a split-brain patient. *Brain Cogn.* 55 303–306. 10.1016/j.bandc.2004.02.026 15177801

[B21] Gori S. Seitz A. R. Ronconi L. Franceschini S. Facoetti A. (2016). Multiple causal links between magnocellular–dorsal pathway deficit and developmental dyslexia. *Cereb. Cortex* 26 4356–4369. 10.1093/cercor/bhv206 26400914 PMC6317503

[B22] Hari R. Renvall H. (2001). Impaired processing of rapid stimulus sequences in dyslexia. *Trends Cogn. Sci* 5 525–532. 10.1016/S1364-6613(00)01801-5 11728910

[B23] Hilchey M. D. Klein R. M. Satel J. (2014). Returning to “inhibition of return” by dissociating long-term oculomotor IOR from short-term sensory adaptation and other nonoculomotor “inhibitory” cueing effects. *J. Exp. Psychol. Hum. Percept. Perform.* 40 1603–1616. 10.1037/a0036859 24820438

[B24] Holmes J. Adams W. J. (2006). Working memory and children’s mathematical skills: implications for mathematical development and mathematics curricula. *Int. J. Exp. Educ. Psychol.* 26 339–366. 10.1080/01443410500341056

[B25] Jarvis H. L. Gathercole S. E. (2003). Verbal and nonverbal working memory and achievements on national curriculum tests at 11 and 14 years of age. *Educ. Child Psychol.* 20 123–140.

[B26] Klein R. (1988). Inhibitory tagging system facilitates visual search. *Nature* 334 430–431. 10.1038/334430a0 3405288

[B27] Klein R. (2000). Inhibition of return. *Trends Cogn. Sci.* 4 138–147. 10.1016/S1364-6613(00)01452-2 10740278

[B28] Klein R. M. MacInnes J. W. (1999). Inhibition of return is a foraging facilitator in visual search. *Psychol. Sci.* 10 346–352. 10.1111/1467-9280.00166

[B29] Koch C. Ullman S. (1985). Shifts in selective visual attention: towards the underlying neural circuitry. *Hum. Neurobiol.* 4 219–227. 10.1007/978-94-009-3833-5_5 3836989

[B30] Krause M. B. (2015). Pay attention!: sluggish multisensory attentional shifting as a core deficit in developmental dyslexia. *Dyslexia* 21 285–303. 10.1002/dys.1505 26338085

[B31] Kucian K. Loenneker T. Dietrich T. Dosch M. Martin E. von Aster M. (2006). Impaired neural networks for approximate calculation in dyscalculic children: a functional MRI study. *Behav. Brain Funct.* 2:31. 10.1186/1744-9081-2-31 16953876 PMC1574332

[B32] Langley L. K. Friesen C. K. Saville A. L. Ciernia A. T. (2011). Timing of reflexive visuospatial orienting in young, young-old, and old-old adults. *Attent. Percept. Psychophys.* 73 1546–1561. 10.3758/s13414-011-0108-8 21394555 PMC3387807

[B33] Lepsien J. Pollmann S. (2002). Covert reorienting and inhibition of return: an event-related fMRI study. *J. Cogn. Neurosci.* 14 127–144. 10.1162/089892902317236795 11970781

[B34] Lewis C. Hitch G. J. Walker P. (1994). The prevalence of specific arithmetic difficulties and specific reading difficulties in 9- to 10-year-old boys and girls. *J. Child Psychol. Psychiatry* 35 283–292. 10.1111/j.1469-7610.1994.tb01162.x 8188799

[B35] Li D. Chen G. (1989). *Combined Raven’s Test (CRT): Chinese Revised Version*. Shanghai: East China Normal University.

[B36] Li T. Wang L. Huang W. Zhen Y. Zhong C. Qu Z. (2018). Onset time of inhibition of return is a promising index for assessing cognitive functions in older adults. *The J. Gerontol. Ser. B Psychol. Sci. Soc. Sci.* 10..1093/geronb/gby070 [Epub ahead of print]. 29878278

[B37] Lupiáñez J. Milán E. G. Tornay F. J. Madrid E. Tudela P. (1997). Does IOR occur in discrimination tasks? Yes, it does, but later. *Percept. Psychophys.* 59 1241–1254. 10.3758/BF03214211 9401458

[B38] Lupiáñez J. Milliken B. Solano C. Weaver B. Tipper S. P. (2001). On the strategic modulation of the time course of facilitation and inhibition of return. *Q. J. Exp. Psychol. A* 54 753–773. 10.1080/713755990 11548033

[B39] MacPherson A. C. Klein R. M. Moore C. (2003). Inhibition of return in children and adolescents. *J. Exp. Child Psychol.* 85 337–351. 10.1016/S0022-0965(03)00104-8 12906846

[B40] Marois R. Chun M. M. Gore C. J. (2000). Neural correlates of the attentional blink. *Neuron* 28 299–308. 10.1016/S0896-6273(00)00104-5 11087002

[B41] McLean J. F. Hitch G. (1999). Working memory impairments in children with specific arithmetic learning difficulties. *J. Exp. Child Psychol.* 74 240–260. 10.1006/jecp.1999.2516 10527556

[B42] Muiños M. Palmero F. Ballesteros S. (2016). Peripheral vision, perceptual asymmetries and visuospatial attention in young, young-old and oldest-old adults. *Exp. Gerontol.* 75 30–36. 10.1016/j.exger.2015.12.006 26702735

[B43] Mussolin C. De Volder A. Grandin C. Schlögel X. Nassogne M.-C. Noël M.-P. (2010). Neural correlates of symbolic number comparison in developmental dyscalculia. *J. Cogn. Neurosci.* 22 860–874. 10.1162/jocn.2009.21237 19366284

[B44] Passolunghi M. C. Mammarella I. C. (2012). Selective spatial working memoryimpairment in a group of children with mathematics learning disabilities and poorproblem-solving skills. *J. Lean. Disabil.* 45 341–350. 10.1177/0022219411400746 21444930

[B45] Posner M. I. (1980). Orienting of attention. *Q. J. Exp. Psychol.* 32 3–25. 10.1080/00335558008248231 7367577

[B46] Posner M. I. Cohen Y. (1984). “Components of visual orienting,” in *Attention and Performance X: Control of Language Processes* eds Bouma H. Bouwhuis D. G. (Hillsadale, NJ: Erlbaum) 531–556.

[B47] Pratt J. Fischer M. H. (2002). Examining the role of the fixation cue in inhibition of return. *Can. J. Exp. Psychol.* 56 294–301. 10.1037/h0087405 12491653

[B48] Price G. R. Holloway I. Räsänen P. Vesterinen M. Ansari D. (2007). Impaired parietal magnitude processing in developmental dyscalculia. *Curr. Biol.* 17 1042–1043. 10.1016/j.cub.2007.10.013 18088583

[B49] Samuel G. A. Kat D. (2003). Inhibition of return: a graphical meta-analysis of its time course and an empirical test of its temporal and spatial properties. *Psychon. Bull. Rev.* 10 897–906. 10.3758/BF03196550 15000537

[B50] Shalev R. S. Auerbach J. Gross-Tsur V. (1995). Developmental dyscalculia behavioral and attentional aspects: a research note. *J. Child Psychol. Psychiatry* 36 1261–1268. 10.1111/j.1469-7610.1995.tb01369.x 8847384

[B51] Swanson H. L. Sachse-Lee C. (2001). Mathematical problem solving and working memory in children with learning disabilities: both executive and phonological processes are important. *J. Exp. Child Psychol.* 79 294–321. 10.1006/jecp.2000.2587 11394931

[B52] Theeuwes J. Kramer A. F. Hahn S. Irwin D. E. (1998). Our eyes do not always go where we want them to go: capture of the eyes by new objects. *Psychol. Sci.* 9 379–385. 10.1111/1467-9280.00071

[B53] Toll S. W. Van der Ven S. H. Kroesbergen E. H. Van Luit J. E. (2011). Executive functions as predictors of math learning disabilities. *J. Learn. Disabil.* 44 521–532. 10.1177/0022219410387302 21177978

[B54] Trick L. M. Enns J. T. (1998). Lifespan changes in attention: the visual search task. *Cogn. Dev.* 13 369–386. 10.1016/S0885-2014(98)90016-8

[B55] Van Selst M. Jolicoeur P. (1994). A solution to the effect of sample size on outlier elimination. *Q. J. Exp. Psychol.* 47 631–650. 10.1080/14640749408401131

[B56] von Aster M. G. Shalev R. S. (2007). Number development and developmental dyscalculia. *Dev. Med. Child Neurol.* 49 868–873. 10.1111/j.1469-8749.2007.00868.x 17979867

