# Supplementary material for: Children With Mathematical Learning Difficulties Are Sluggish in Disengaging Attention
Source: Front Psychol. 2019 May 9;10:932. doi: 10.3389/fpsyg.2019.00932 (PMC6520633; doi:10.3389/fpsyg.2019.00932)
Supplement: Supplementary file 1 [file Data_Sheet_1.PDF]

Supplemental material for

"***Children with Mathematical Learning Difficulties are Sluggish in Disengaging Attention***" by Zhang X., Fu W., Xue L., Zhao J., & Wang Z.

Experiment S1

The reviewers of this paper raised some concerns about statistical power, as the sample size in Experiment 1 was relatively small, 15 and 14 for the TD and MLD groups, respectively. In an effort to address this issue, a second set of data was collected from a different school. The screening protocol for MLD and the experimental methods were exactly the same as Experiment 1. For convenience, we will refer to this second set of data as Experiment S1. We did not report this set of data in the main manuscript because it was collected about 1.5 years later, in a country where the socio-economic structure is rapidly changing. Combining the data from Experiments 1 and S1 would increase the sample size to 35 and 34 for the TD and MLD groups. Here we report a combined analysis of the RTs to address the statistical concerns. The overall pattern of the results from this combined analysis was the same as that reported in the main manuscript.

Participants

For Experiment S1, a total of 520 third-grade children from a local elementary school were screened for MLD. All children were native Mandarin speakers and they all had normal or corrected-to-normal visual acuity. The school records show that none of them were socio-culturally disadvantaged or had behavioral or neuropsychological conditions. They were naive with regard to the purpose of the experiments and had not participated in other psychological studies.

The screening criteria for MLD was the same as Experiment 1. This gave us an additional 20 subjects for both the TD and MLD group. The age, non-verbal IQ, and math scores of these subjects are presented in Table S1. The TD group was age-matched to the MLD group, $t(38)=1.22$, $p = .23$, Cohen's $d = 0.41$. The MLD group

scored lower on the non-verbal intelligence test, $t(38) = 6.69$, $p < .001$, Cohen's d = 2.12, but their non-verbal IQ was all in the normal range. The scores on the most recent math exam were lower in MLD group compared to that in the TD group, $t(38) = 13.85$, $p < .001$, Cohen's $d = 4.38$.

Table S1. *Age, non-verbal IQ, and mathematical scores of the MLD and TD children in Experiment S1.*

	N	Age (years)	Non-verbal IQ	Math score
TD	20	9.13 (0.31)	123.10 (8.25)	94.38 (2.05)
MLD	20	9.28 (0.41)	105.10 (8.76)	57.33 (11.79)

Data preprocessing for Experiment S1

The data processing protocol was exactly the same as Experiment 1. As in Experiment 1, 25% of the trials were catch trials. An ANOVA on the false alarm rates revealed no significant effect for group (TD vs MLD), $F(1,38) = 1.453$, p = 0.236, , $\eta_p^2 = 0.037$. The false alarm rates for the MLD and TD children were 4.06% and 2.50%, respectively. The target was rarely missed. The miss rates for the MLD and TD children were 0.63 % and 0.72%, respectively. An ANOVA on the miss rates revealed no significant effect, all $F < 1$.

Eye movements were detected on 18.68% and 16.84% of the trials (including catch trials) in the MLD and TD children, respectively. An ANOVA on the proportions of trials excluded due to eye movements, with variables group (MLD vs. TD) and CTOA, revealed no significant effect, all $F < 1$.

The RTs from the non-catch trials were cleaned based on the number of trials in each experimental cell of each participant, following the same protocol of Experiment 1. This procedure excluded only a small proportion of the trials, 3.57%, 3.17% for the MLD and TD children, respectively. An ANOVA on the proportion of trials excluded due to this cleansing procedure revealed no significant effect, all $F < 2.03$.

Combined RT analysis

The RTs from Experiments 1 and S1 was combined to perform an ANOVA, with variables group (TD vs MLD), cueing (valid vs invalid), and CTOA (100, 200, 400, or 800 ms). The results revealed significant main effects for group, $F(1, 67) = 13.21$, $p < .001$, $\eta_p^2 = 0.17$, and CTOA, $F(3, 201) = 35.57$, $p < 0.001$, $\eta_p^2 = 0.35$. The main effect of cueing was not significant, $F < 1$, but 2-way interactions occurred between group and cueing, $F(1, 67) = 9.98$, $p = .002$, $\eta_p^2 = 0.13$, and between CTOA and cueing, $F(3, 201) = 14.32$, $p < .001$, $\eta_p^2 = 0.18$. The main effect of group was significant because the RTs were generally longer in the MLD children. The main effect of CTOA occurred because the temporal expectation for the target strengthened at longer CTOAs, as in other cueing tasks. The interaction between CTOA and cueing reflects the prototypical time course of cueing effects, i.e., early facilitation followed by IOR. The interaction between group and cueing occurred because the cueing effect in the TD group was overall more positive and thus, the cueing effect crossed 0-ms at a shorter CTOA. Consider also the non-significant 3-way interaction, the time course of cueing effect in the MLD group can be regarded as a global shift towards "facilitation" (see Figure S1). An ANCOVA was also performed on the RTs, with the non-verbal IQ as a covariate. The overall pattern of the results was the same as that reported here.

Planned comparisons revealed significant facilitation effect at the 100-ms CTOA, $t(34) = 2.13$, $p = .04$, $d_z = 0.19$, and IOR at the 400-ms and 800-ms CTOAs, all $t > 5.3$, all p's $< .001$, all $d_z > 0.46$, in the TD group. In the MLD group, significant facilitation was observed at both the 100-ms CTOA, $t(33) = 2.30$, $p = .03$, $d_z = 0.22$, and the 200-ms CTOA, $t(33) = 2.895$, $p = .007$, $d_z = 0.25$, however, no IOR was observed at longer CTOAs.

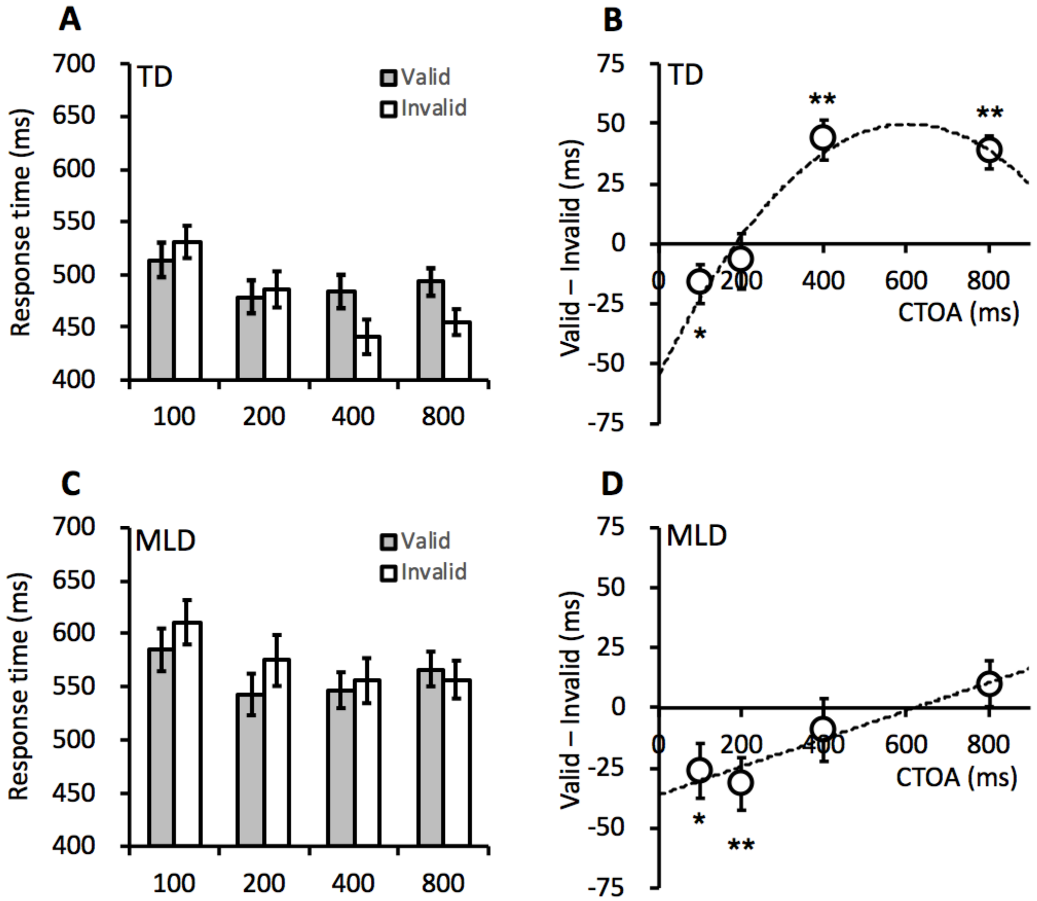

Figure S1. *(A) & (C): Mean target RTs for the TD and MLD children in the combined analysis. (B) & (D): The cueing effects for the TD and MLD children. Dashed lines are polynomial fittings of the cueing effects against CTOA. CTOA: the time interval (in milliseconds) between cue onset and target onset. Error bars denote ± 1 SEM. *p < 0.05, **p < 0.01.*
